# Cluh plays a pivotal role during adipogenesis by regulating the activity of mitochondria

**DOI:** 10.1038/s41598-019-43410-4

**Published:** 2019-05-02

**Authors:** Eugene Cho, Wonhee Jung, Hyun-Yoo Joo, Eun-Ran Park, Mi-Yeon Kim, Su-Bin Kim, Kwang Seok Kim, Young Bin Lim, Kee Ho Lee, Hyun Jin Shin

**Affiliations:** 1 Team of Radiation Convergence Research, Korea Institute of Radiological & Medical Sciences, Seoul, Korea; 2 Division of Radiation Biomedical Research, Korea Institute of Radiological & Medical Sciences, Seoul, Korea

**Keywords:** Lipid signalling, Fat metabolism, Mitochondria

## Abstract

Cluh is a cytosolic protein that is known to specifically bind the mRNAs of nuclear-encoded mitochondrial proteins and play critical roles in mitochondrial biogenesis. Here, we report the role of Cluh in adipogenesis. Our study shows that mRNA expression of Cluh is stimulated during adipogenesis, and that cAMP/Creb signalling increases its transcription. Cluh depletion impaired proper adipocyte differentiation, with reductions seen in lipid droplets and adipogenic marker gene expression. Interestingly, the inductions of the brown adipocyte-specific genes, *Ucp1*, *Cidea* and *Cox7a1*, are severely blocked by Cluh depletion during brown adipogenesis. Mitochondrial respiration and the stability of mRNAs encoding mitochondrial proteins are reduced by Cluh depletion during brown adipogenesis. These results suggest that Cluh, which is induced during adipogenesis, promotes the post-transcriptional regulation of mitochondrial proteins and supports differentiation.

## Introduction

Mitochondrial biogenesis is up-regulated during adipocyte differentiation and promotes this process^1,2^. The mitochondrial density increases and structural and biochemical alterations are seen in both brown and white adipose cells^3^. Mitochondrial biogenesis plays a pivotal role in maintaining metabolic homeostasis in adipose tissue. Wilson-Fritch *et al*. had reported 20–30-fold increase in concentration of numerous mitochondrial proteins during the differentiation of 3T3-L1 cells^4^. However, increased levels of these proteins were not accounted for by changes in transcription; the post-transcriptional regulatory mechanism of adipogenesis is not yet well understood.

CLUH (clustered mitochondria homolog) was first identified as a contributing factor in the determination of mitochondrial distribution in D. discoideum^5^. A defect in CLUH induces mitochondrial clustering and reduces the levels of mitochondrial proteins, resulting in oxidative phosphorylation defects in murine embryonic fibroblasts (MEFs) and human cancer cells^5–8^. In Drosophila, Clu, a homolog of CLUH, was found to associate with mitochondrial outer membrane protein^9^. Recently, hCLUH was reported as the first RNA-binding protein (RBP) that binds to the mRNAs that encode mitochondrial proteins^7^.

In the present study, we investigated the role of Cluh in adipocyte differentiation. *Cluh* was found to be transcriptionally activated during adipogenesis, its transcription being regulated by cAMP/Creb signalling. Depletion of Cluh decreased lipid droplets and reduced the transcription of adipocyte marker proteins. In particular, the expression of brown adipocyte marker genes was severely impaired by depletion of CLUH from C3H10T1/2 cells; such cells exhibit mitochondrial dysfunction during adipogenesis, related to the impaired expression of mitochondrial mRNAs. The reduction of transcripts in CLUH-depleted adipocytes was associated with a decrease in mRNA stability.

## Results

### Transcription of CLUH is activated during adipocyte differentiation

**Figure 1:**
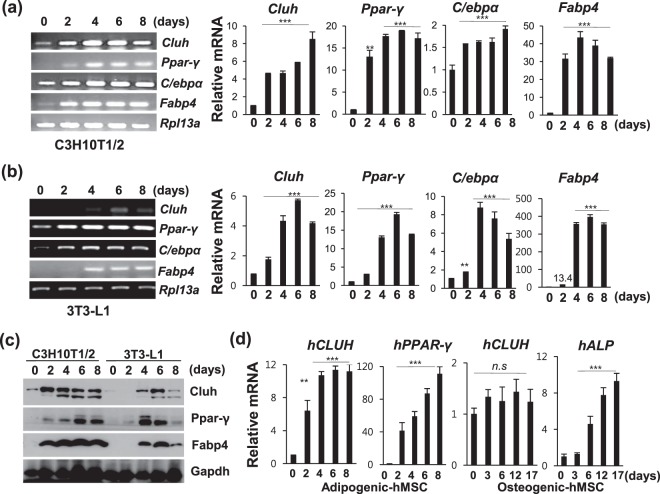
Cluh is increased during adipogenesis. (**a**,**b**) Expression of *Cluh* was analyzed in C3H10T1/2 cells (**a**) and 3T3-L1 cells (**b**) at the indicated times after adipocyte induction, using RT-PCR and quantitative RT-PCR. Differentiation was confirmed by analyzing the adipogenesis markers, *Ppar-γ*, *FABP4*, and *C/ebpα*. Full-length gels are presented in Supplementary Fig. 2a,b. (**c**) Increase of Cluh protein levels during adipogenesis was analyzed by western blotting. Differentiation was confirmed by detection of the Ppar-γ protein. A full-length blot is presented in Supplementary Fig. 3. (**d**) Transcription of human *CLUH* during adipogenesis and osteogenesis was assessed using hMSCs. A dipogenesis or osteogenesis in hMSCs was confirmed by quantifying the transcription of *PPAR- γ* or *ALP*, respectively. In (**a**–**d**), all data are representative of three (**c**,**d**) or five (**a**,**b**) independent experiments. For quantitative RT-PCR, the Ct values of target mRNAs were normalized to those of *Rpl13a* for murine cells and *B2M* for human cells. Results were calculated using the ΔΔCt method and presented as the means ± SD of triplicate reactions. * indicates *p* < *0*.*05*, ** indicates *p* < *0*.*01*, and *** indicates *p* < *0*.*001*.

Since CLUH has been known to be a post-transcriptional regulator of mitochondrial protein, we hypothesized that it may play a crucial role in adipocytes, where the activity of mitochondria is increased^1–4^. To investigate the role of CLUH in adipocytes, we first examined its expression during adipogenesis. As *in-vitro* models for adipocyte differentiation, we selected murine cell lines that have been widely used in studying adipose tissue: 3T3-L1 cells represent a pre-adipose cell line for white adipocyte differentiation, and C3H10T1/2 cells represent a mesenchymal stem cell line for brown adipocyte differentiation. Differentiation protocol is illustrated in Supp. Fig. 1a. We validated our differentiation protocols for the two cell lines (Supp. Fig. 1a) by RT-PCR, western blotting, and Oil-Red O staining (Fig. 1a–c, Supp. Fig. 1b).

*Cluh* mRNA was found to increase during adipogenesis in C3H10T1/2 and 3T3-L1 cells (Fig. 1a,b, Supp. Fig. 2), beginning 2 days after adipogenic induction. This represents the clonal expansion stage, which is a relatively early stage of differentiation. The expression of *Cluh* during adipogenesis in murine pre-adipocytes was increased by approximately 7–10 fold, and the Cluh protein levels were also increased during adipogenesis in C3H10T1/2 and 3T3-L1 cells (Fig. 1c, Supp. Fig. 3).

To verify whether human *CLUH* also induced during adipogenesis, human mesenchymal stem cells (hMCSs) were differentiated into adipocytes and the expression of *CLUH* were analyzed. As shown in Fig. 1d, transcriptional level was enhanced up to approximately 11-fold. Since osteogenic and adipogenic lineages share common features, such as transcription pathways or increase of mitochondrial biogenesis^10^, we examined whether *CLUH* is also increased in hMSCs differentiating into osteocytes. *CLUH* was not found to increase in osteogenic differentiating cells, whereas the osteogenic marker, ALP, was noticeably induced (Fig. 1d).

### Depletion of cluh impairs adipogenic differentiation

**Figure 2:**
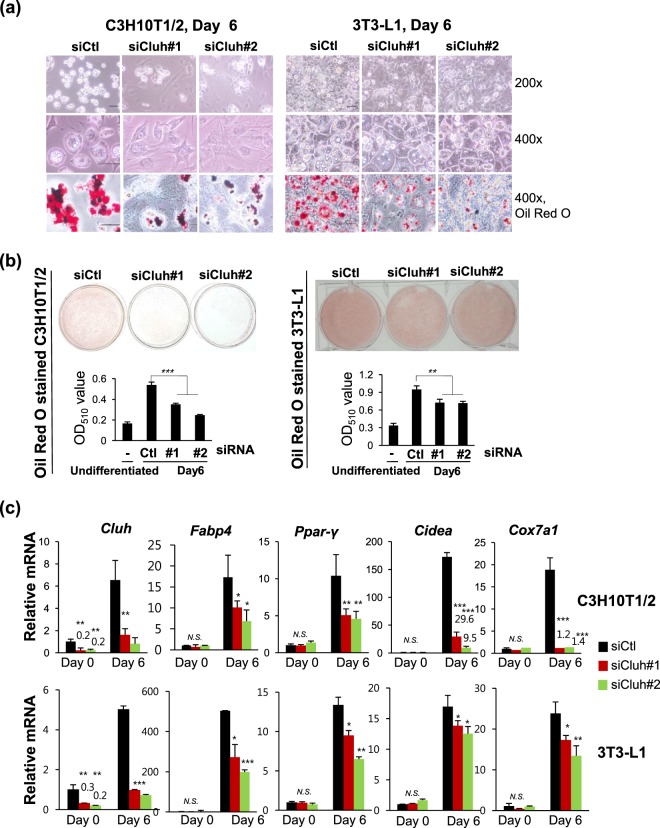
Cluh is essential for adipocyte differentiation. (**a**) C3H10T1/2 and 3T3-L1 cells were transfected with Cluh siRNAs, differentiated to adipocytes for 6 days, visualized under light microscopy, and photographed at magnification 200× and 400×. Cell morphology and lipid droplet formation of Cluh-depleted cells were compared to those of control siRNA-transfected cells. In the lower panel, the cells were stained with Oil Red O and photographed under light microscopy at magnification 400x. Scale bar: 50 μm. (**b**) 3T3-L1 and C3H10T1/2 cells were transfected with siRNAs, differentiated for 6 days, and stained with Oil Red O. The intensity of Oil Red O staining was first captured by photography and then quantified by extracting the dissolved stain and measuring the optical density (O.D.) at 510 nm. The results are representative of at least three independent experiments, and O.D. measurements were performed in triplicate. (**c**) Quantitative RT-PCR analysis was performed to validate the depletion of *Cluh* and confirm adipogenic differentiation (*Fabp4* and *Ppar-γ*) in siRNA-transfected and differentiated cells. Brown adipogenic differentiation was evaluated by analyzing the expression of brown adipocyte marker genes, *Cidea* and *Cox7a1*. At least three independent experiments were performed, and data are presented as the average of triplicate reactions in a representative experiment. Ct values of target mRNAs were normalized with respect to that of *Rpl13a*. * indicates *p* < *0*.*05*, ** indicates *p* < *0*.*01*, and *** indicates *p* < *0*.*001*.

To investigate the role of increased Cluh during adipogenesis, we performed siRNA-mediated knockdown of Cluh in C3H10T1/2 and 3T3-L1 cells, which were then differentiated into adipocytes and assessed for phenotypes related to adipogenesis (cell morphology, lipid droplets and adipocyte-specific gene expression). As shown in Fig. 2a, differentiated control cells took on the rounded features characteristic of differentiated adipocytes, whereas the Cluh-depleted cells, especially in the case of C3H10T1/2 cell line, maintained their fibroblast-like morphology. Moreover, number and size of lipid droplets (organelles in which lipids accumulate in differentiating adipocytes) was significantly reduced by Cluh depletion (Fig. 2a), as was the intensity of Oil Red O staining, which can be used to monitor lipid droplet accumulation (Fig. 2a,b). This apparent CLUH depletion-induced reduction in lipid droplets was quantitatively confirmed hereafter, by measuring the optical density of dissolved Oil Red O (Fig. 2b).

We next analyzed the transcription of adipose cell biomarkers, *Ppar-γ* and *Fabp4*, and found that they were reduced by Cluh depletion in cells undergoing adipogenesis (Fig. 2c). Interestingly, during the adipogenesis of C3H10T1/2 cells, expression levels of the brown adipocyte marker genes, *Cidea* and *Cox7a1*, were severely reduced by Cluh depletion (6~18 fold and 14 fold, respectively), whereas those of *Fabp4* and *Ppar-γ* decreased by only about 2 fold.

### Cluh is up-regulated by cAMP/Pka/Creb signaling, but not by Ppar-γ or C/EBP α/β

To investigate the machinery that controls the transcriptional induction of *Cluh* during adipogenesis, we examined the potential involvement of master regulators of adipogenic differentiation, including the transcription factors, *Ppar-γ*, *C/ebpα* and *C/ebpβ*, and cAMP signaling. We added cell line, NIH-3T3, to find a key adipogenic transcription factor that regulates Cluh directly, regardless of the conditions related to adipogenic differentiation.

**Figure 3:**
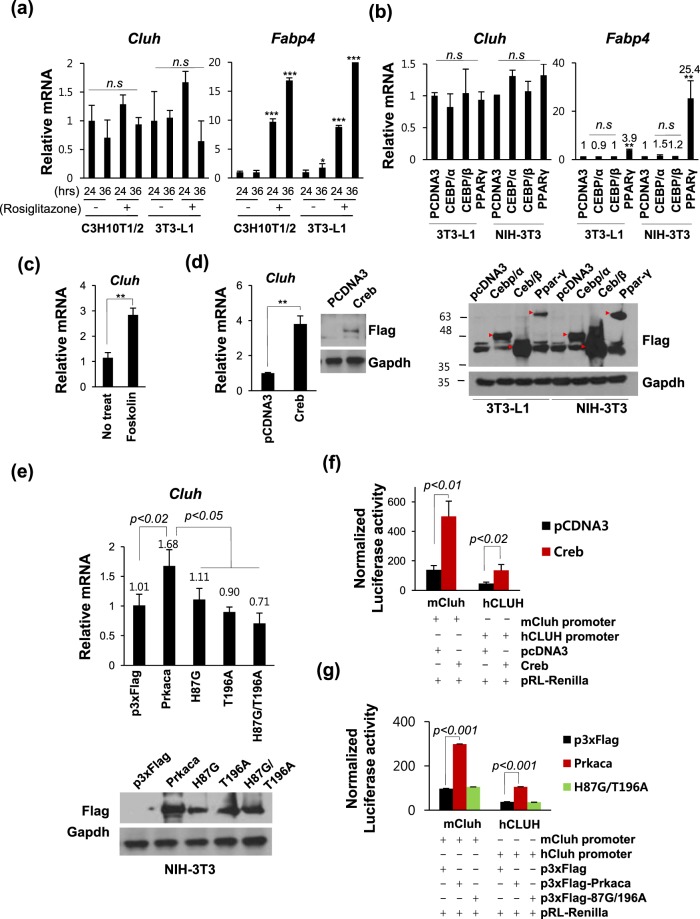
Transcription of *Cluh* is increased by cAMP/Creb, not by Ppar-γ. (**a**) C3H10T1/2 and 3T3-L1 cells were treated with rosiglitazone (5 µg/ml) without differentiation and the mRNA levels of *Cluh* and *Fabp4* were analyzed with real time PCR. (**b**) Plasmids encoding C/ebpα, C/ebpβ, and Ppar-γ were transfected into exponentially growing 3T3-L1 and NIH-3T3 cells. After 24 h, the mRNA levels of *Cluh* and *FABP4* were analyzed with real time PCR (upper panel). The expression of transfected plasmids was confirmed with western blotting using Flag antibody (lower panel). (**c**) NIH-3T3 cells were treated with forskolin for 6 h and the mRNA levels of Cluh were analyzed with real time PCR. (**d**,**e**) NIH-3T3 cells were transfected with plasmids encoding Creb (**d**), Prkaca, or its catalytic site mutants (**e**). After 24 h, the mRNA levels of *Cluh* were analyzed with real time PCR. Expression of transfected plasmids was confirmed with western blotting using Flag antibody (d; right panel, e; lower panel). (**f**,**g**) The activities of *mCluh* and *hCLUH* promoters were enhanced by Creb and Prkaca. Cells were co-transfected with pGL3-basic, pGL3-mCLUH promoter, or pGL3-hCLUH promoter plus Creb- (**f**) or Prkaca**-**(**g**) expressing plasmids or their backbone vectors. Firefly luciferase activity was normalized to that of Renilla luciferase and fold induction was plotted with respect to the normalized luciferase activity of pGL3-Basic vector-transfected cells. In (**a**–**g**), results presented are representative of three independent experiments, all performed in non-differentiated conditions. In (**a**,**b**), the average Ct value was calculated from triplicate quantitative RT-PCRs; in (**c**–**g**), experiments were performed in triplicate from the cell-seeding stage. * indicates *p* < *0*.*05*, ** indicates *p* < *0*.*01*, and *** indicates *p* < *0*.*001*.

First, we treated with Ppar-γ agonist, rosiglitazone to analyze the effect of Ppar-γ activation on the transcription of Cluh. Transcription levels of *Cluh* were not increased by the activation of Ppar-γ in 3T3-L1 and NIH-3T3 cells whereas *Fabp4* was increased (Fig. 3a). To validate the effect of C/ebpα, C/ebpβ and Ppar-γ on the transcription of *Cluh*, we transfected cells with plasmids encoding the various regulators and confirmed their protein expression by western blot analysis (Fig. 3b lower panel, Supp. Fig. 4a). Ppar-γ overexpression increased *Fabp4* as reported (Fig. 3b, upper panel)^11^, but did not affect the transcription of *Cluh*. Effect of Ppar-γ on the transcription of *Fabp4* was greater in NIH-3T3 cells due to the higher transfection efficiency (Fig. 3b lower panel). Although ectopic protein expression was higher than Ppar-γ, C/ebpα, and C/ebpβ also did not affect the transcription of *Cluh* and *Fabp4* significantly (Fig. 3b).

To test whether cAMP signaling regulates the transcription of *Cluh*, we treated NIH-3T3 cells with forskolin for 6 hours and found that the transcription of *Cluh* to be three-fold higher after post-treatment (Fig. 3c). We also observed that overexpression of the cAMP-responsive transcription factor, Creb, increased the transcription of *Cluh* (Fig. 3d). Since the cAMP signaling pathway activates Creb through protein kinase A (Pka), we examined whether Pka could regulate *Cluh* transcription. Toward this end, we used plasmids encoding Prkaca (the catalytic subunit alpha of Pka) and its kinase-defective mutants H87G, T196A, and H87G/T196A^12^. As shown in Fig. 3e, Prkaca wild type increased the transcription of Cluh while all three mutant constructs did not. The plasmid-driven expression of Creb and Prkaca were confirmed by western blot analysis (Fig. 3d, right panel; Fig. 3e, lower panel; Supp. Fig. 4b).

To determine whether Pka and/or Creb regulate the transcription of *Cluh* by modulating its promoter activity, we performed luciferase assays using the promoters of *mCluh* and *hCLUH* (Fig. 3f,g). The promoter activity of *CLUH* was 110-fold (in mouse) and 40-fold (in human) greater than that of the promoter-less mock vector, pGL3-Basic The activities of both human and mouse promoters were increased by the overexpression of Creb (3.6- and 3- fold, respectively; Fig. 3f) and Prkaca (3- and 2.8- fold, respectively; Fig. 3g). The catalytic-site mutant of Prkaca did not affect the activity of either promoter (Fig. 3g). We also confirmed the increase of Cluh protein by the overexpression of Creb and Prkaca in 3T3-L1 cells using western blotting (Supp. Fig. 4c).

### Cluh is required for mitochondrial respiration during adipogenesis

**Figure 4:**
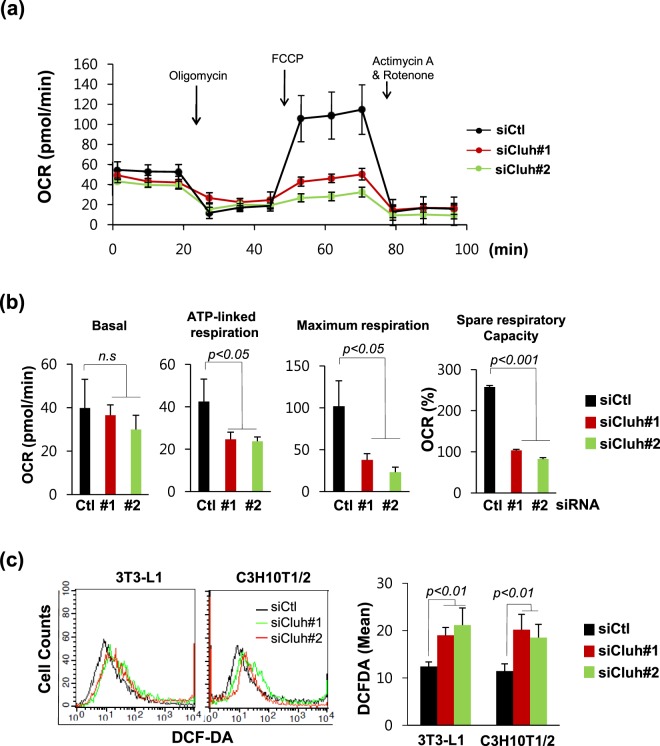
Cluh depletion impairs mitochondrial respiration during adipogenesis. (**a**,**b**) The oxygen consumption rate (OCR) in Cluh-depleted C3H10T1/2 cells was analyzed using a Seahorse XF-24 analyzer. Data are presented as the average of triplicate measurements and error bars indicate the standard deviation. (**a**) Mitochondrial respiration, reflected by the OCR level, was detected in non-differentiated control cells (ND) and siRNA-transfected and differentiated cells (siCtl, siCluh#2). (**b**) Rates of basal respiration, ATP-linked respiration, maximal respiratory capacity, and spare respiratory capacity were quantified by normalization of OCR levels to total protein levels obtained from O.D. values. Duplicate independent OCR experiments were performed and average of triplicate was obtained for each sample. (**c**) Intracellular ROS levels in Cluh-depleted and differentiated 3T3-L1 and C3H10T1/2 cells were analysed by flow cytometric detection of DCFDA fluorescence. The average was obtained from three independent experiments.

We examined whether the function of Cluh during adipogenesis and in adipocytes was related to its known ability to regulate mitochondrial biogenesis^7,13^. We analyzed mitochondrial activity in Cluh-depleted and adipocyte differentiated (at day 4) C3H10T1/2 cells using a Seahorse XF analyzer to determine the indicator of mitochondrial respiration, Oxygen Consumption Rate (OCR) (Fig. 4a). Depletion of Cluh decreased ATP-linked respiration, maximum respiration rate and spare respiratory capacity (Fig. 4b). The maximal OCR was not increased relative to the basal OCR and the spare respiratory capacity was severely reduced compared to that of control cells. These results imply that the mitochondria in Cluh-depleted cells were already working at their maximal capacity to compensate for the defective mitochondrial respiration. We further analyzed mitochondrial dysfunction by measuring ROS, and found it to be increased upon Cluh depletion during adipogenesis in both 3T3-L1 and C3H10T1/2 cells (Fig. 4c).

### Cluh regulates mRNA stability of mitochondrial proteins during adipogenesis

**Figure 5:**
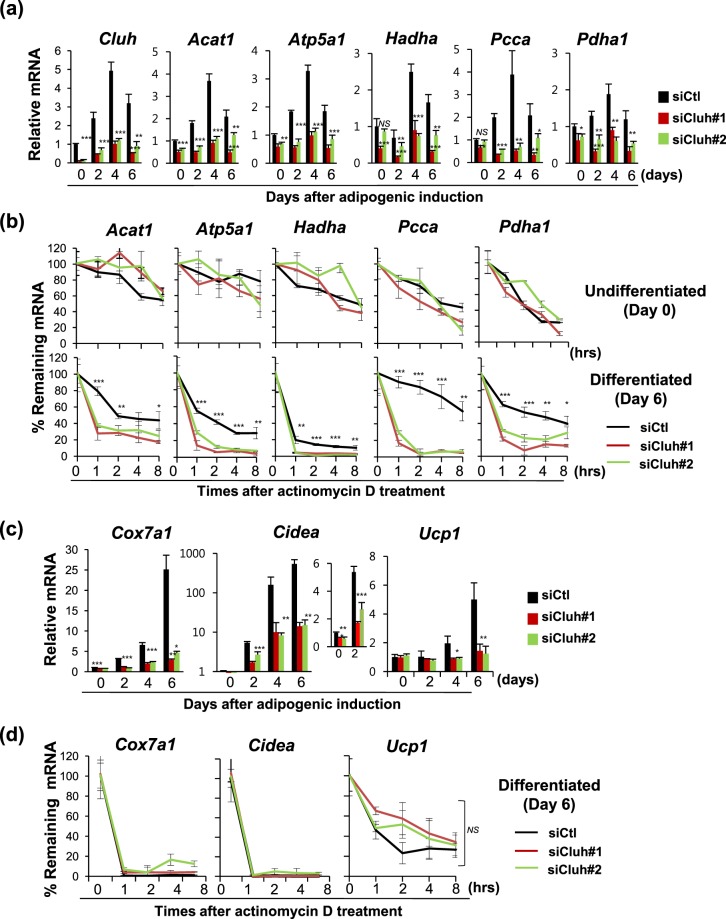
Cluh stabilizes the mRNA of mitochondrial genes, especially during adipogenesis. (**a**) C3H10T1/2 cells were transfected with siRNA at day −1 and adipogenesis was induced. Cells were harvested at days 0, 2, 4, and 6, and the mRNA levels of mitochondrial genes were quantified. Ct values were normalized to those of Rpl13a. (**b**) Effect of Cluh depletion on mRNA stability. siRNA-transfected and/or differentiated C3H10T1/2 cells were treated with actinomycin D (2 μg/ml), harvested at the indicated times, and mRNAs quantified with quantitative RT-PCR. (**c**,**d**) mRNA transcript levels and mRNA stability, of genes induced in brown adipocytes, were quantified from the cDNA samples described in A and C. Quantitative RT-PCR results were obtained from three independent experiments and calculated as the average of triplicate reactions per experiment. * indicates *p* < *0*.*05*, ** indicates *p* < *0*.*01*, and *** indicates *p* < *0*.*001*.mRNA stability of mitochondrial genes in undifferentiated cells (**b**) and those of Cox7a1 and Cidea (**d**) were not statistically processed since their stability was not definitely decreased by Cluh depletion.

Since Cluh is known to control the stability of mRNA and translation of many mitochondrial proteins^13^, we investigated whether it could control the nuclear-encoded mitochondrial genes, *Acat1*, *Atp5a1*, *Hadha*, *Pcca*, and *Pdha1*, during adipogenesis. These genes had been previously selected as representative nuclear-encoded mitochondrial genes from among hundreds of screened candidate transcripts that may bind to CLUH^7^, and the ability of CLUH to stabilize their mRNAs has been verified^13^. RNA levels of all tested genes were found to have increased during adipogenesis in C3H10T1/2 cells, and these increases were inhibited by depletion of Cluh (Fig. 5a). Cluh depletion had greater effect on the transcript levels during adipogenesis compared to those observed prior to differentiation (day 0).

To address whether the reduction of mRNA levels in Cluh-depleted cells was related to post-transcriptional regulation of RNA stability, we measured mRNA decay in siRNA-transfected cells that had differentiated into adipocytes (day 6) by treatment of actinomycin D. As shown in Fig. 5b, the mRNAs of all tested genes were destabilized by depletion of Cluh in adipose-differentiated C3H10T1/2 cells (Fig. 5b, shown in log scale in Supp. Fig. 5).

Since Cluh depletion clearly reduced the expression levels of brown adipocyte-related genes (Fig. 2c), we next explored whether the mRNA stabilities of *Cox7a1*, *Cidea* and *Ucp1* were regulated by Cluh. Despite the transcripts being severely reduced by Cluh depletion (Fig. 5c), their mRNA stabilities were not significantly reduced (Fig. 5d, shown in log scale in Supp. Fig. 5).

## Discussion

Mitochondria play a crucial metabolic energy-consuming role in adipose tissues. To reduce obesity and improve insulin sensitivity, adipose and other tissues must enhance their mitochondrial oxidation rates, and hence increase energy expenditure. Several previous had reports shown that the mitochondrial function of adipocytes is reduced in metabolic diseases including diabetes, hepatosteatosis, obesity, and cardiovascular complications^14,15^. Mitochondrial functions such as mitochondrial oxidative capacity, mitochondrial membrane potential, and citrate synthase activity were found to be lower in the adipocytes of obese patients compared to that in control group^16,17^. These findings suggest that proteins related to mitochondrial function in adipocytes could be potential targets for treating adipose-related metabolic diseases.

In order to explore the genes that play key roles in adipogenesis, we screened for adipogenesis-induced genes. Amongst the identified genes, we focused on CLUH, since it was known to specifically function as a post-transcriptional regulator in mitochondria. Here, we report that Cluh is up-regulated in both white and brown adipose cells, and plays a crucial role during adipocyte differentiation. Cluh depletion was found to impair lipid droplet formation, proper adipocyte differentiation, capacity of mitochondrial respiration, and stability of genes encoding mitochondrial proteins. We observed lipid droplets as a measure of adipogenic differentiation. Reduction of lipid droplets in Cluh-depleted adipogenic cell differentiation reflects improper differentiation. A recent study has reported that lipid droplets are increased in liver tissue of Cluh-deficient mice due to the metabolic dysfunction of mitochondria, resulting in inefficient β oxidation^13^. However, the different results may have arisen from the same cause of mitochondrial defect. This defect may lead to defective adipogenesis, thereby reducing lipid droplets, or cause inefficient β oxidation, thereby inducing lipid droplets. The effect of Cluh depletion on lipid droplet in other tissues might be different from our results.

Although this is the first report of the contribution of Cluh in adipogenesis, it also confirms that Cluh does not alter the stability of brown adipocyte-specific genes, such as *Ucp1*, *Cidea*, and *Cox7a1*. Instead, its direct targets were *Acat1*, *Atp5a1*, *Hadha*, *Pcca*, and *Pdha1*, which function in relation to central pathways for mitochondrial respiration, namely fatty acid β-oxidation (FAO) and oxidative phosphorylation (OXPHOS). Mitochondria are particularly active in adipocytes, where they help to maintain the normal metabolic functions such as FAO and OXPHOS. We found these genes were increased during adipogenesis, and mRNAs were stabilized by Cluh especially in adipocytes. Therefore, Cluh may contribute to the maintenance of metabolic homeostasis by supporting the appropriate protein expression pattern and subsequent function of mitochondria, thereby facilitating proper adipogenesis.

To define the mechanism by which Cluh is transcriptionally activated during adipogenesis, we analyzed whether adipogenesis-related core transcription factors regulate the transcription of *Cluh*. Our results indicated that transcription of *Cluh* was activated by Pka/Creb signaling, but not by Ppar-γ or C/EBPα/β. Pka/Creb is an upstream regulator of adipogenesis that is known to control C/EBPβ to activate adipocyte-related genes, such as *C/EBPα*, *KLF5*, *PPAR-γ* and *AP2*^18,19^. cAMP signalling has been reported to play a critical role in brown fat by stimulating the levels of key thermogenic factors, such as UCP1^20,21^. In this study, we uncover a novel role of cAMP signaling, as an upstream regulator of Cluh, during adipogenesis. However, the increased levels of *Cluh* by cAMP/CREB signaling were relatively lower than expected. Changes in transcription machinery or in epigenetics during adipogenesis may cooperatively regulate the transcription of *Cluh*.

Since mitochondria support the differentiation and homeostatic function of adipocytes, their dysfunction has serious consequences^1,2^. Excess white adipose tissue (WAT) undergoes various remodeling process, including hypertrophy, hyperplasia and/or inflammation. Sustained obesity and inflammation lead to abnormality of WAT, causing mitochondrial dysfunction and consequent metabolic diseases^22^. Many studies have focused on the remodeling of WAT into brown-like adipose tissue^23–25^. Here, we report that the transcription of *Cox7a1*, *Cidea*, and *Ucp1*, which are brown-fat-specific genes required for thermogenesis and mitochondrial biogenesis, were significantly reduced by the depletion of Cluh from C3H10T1/2 cells while that of common adipogenesis markers were decreased less than 2 fold. Moreover, Cluh depletion decreased lipid droplets more seriously in C3H10T1/2 cells than in 3T3-L1 cells. Thus, Cluh depletion appears to severely impair proper brown adipogenesis. Mitochondrial function is more critical in BAT than in WAT. For example, UCP1, which is exclusively expressed in BAT and known to play a key role in brown adipogenesis, is present in the mitochondrial inner membrane, its activity being greatly affected by mitochondria itself^26^. The abnormal BAT differentiation upon Cluh depletion could be caused by dysfunction of mitochondria. Together, our results suggest that Cluh is more critical in brown adipocytes than in white adipocytes, and affects the characteristics of differentiating adipocytes. Further studies would be required to fully elucidate the role of Cluh in modulating adipocytes.

In summary, we herein show that Cluh plays a key role in adipogenesis by post-transcriptionally regulating mitochondrial proteins in adipocytes. Our findings suggest that CLUH could be a novel target for modulating metabolic energy consumption, controlling adipocyte differentiation, and/or changing the characteristics of adipocytes.

## Methods

### Cell culture

Mouse 3T3-L1 pre-adipocytes, mouse mesenchymal stem cells (mMSCs), C3H10T1/2, and NIH-3T3 cells were purchased from ATCC (American Type Culture Collection, Manassas, VA) and grown in Dulbecco’s modified Eagle’s medium (DMEM; Welgene, Korea) supplemented with 10% bovine calf serum (BCS; Gibco, Carlsbad, CA) and 1% penicillin-streptomycin at 37 °C in 5% CO_2_. Human mesenchymal stem cells (hMSCs) were purchased from CEFO BIO (CB-ADMSC-001, Korea), and cultured and differentiated in media supplied by the manufacturer.

### Adipocyte differentiation

To induce adipocyte lineage commitment, 3T3-L1 pre-adipocytes were seeded at 8 × 10^4^ cells/well in either six-well plates or 35-mm dishes (day −4). At day −2, the dishes in which cells had reached 100% confluence were selected and incubated further for 2 more days. At day 0, the growth medium was removed and replaced with adipocyte differentiation medium (DMEM supplemented with 10% FBS, 1% antibiotics, 0.5 mM IBMX (Sigma-Aldrich, St. Louis, MO), 1 µM dexamethasone (Sigma-Aldrich), and 10 µg/ml insulin (Sigma-Aldrich)). At day 2, the medium was changed to DMEM supplemented with insulin (10 µg/ml); thereafter, the medium was replaced by the same insulin-supplemented DMEM every 2 days. C3H10T1/2 stem cells were seeded at 5 × 10^4^ cells/well in six-well plates (day −5). The following day (day −4), 50 ng/ml BMP4 (R&D Systems, Minneapolis, MN) was added. Further differentiation was conducted, as described for 3T3-L1 cells. A schematic diagram of the differentiation schedule is presented in Supplementary Fig. 1.

### Oil Red O staining and quantification of differentiation

Differentiated adipocytes on 35-mm dishes were fixed with 10% formalin for 10 min, followed by washing with 60% isopropanol for 5 min. The fixed cells were dried, stained with Oil Red O solution (Sigma-Aldrich) for 1 h, and washed four times with water. For quantification, the retained Oil Red O was dissolved in 5 ml (per dish) isopropanol and its absorbance measured at 510 nm in a plate reader.

### siRNA, plasmids, and transfection

siRNA duplexes were synthesized by Genolution (Korea). The sequences were as follows: Cluh siRNA#1: 5′-GCU UCA AUC CUG ACA UCU U-3′; Cluh siRNA#2: 5′-GGG CAU CAU UGG CAA UGA U-3′; and negative control: 5′-AAU UCU CCG AAC GUG UCA CGU UU-3′. Cells were transfected with siRNA at a final concentration of 50 nM using RNAiMAX (Invitrogen, Carlsbad, CA) in Opti-MEM media (Invitrogen). During adipogenesis, siRNAs were transfected at day −1.

All plasmids used in this study were either purchased from Addgene, or directly cloned, or obtained as gifts. pcDNA flag mPPAR gamma was a gift from Bruce Spiegelman (Addgene plasmid # 8895)^27^. pcDNA-mC/EBPα and pcDNA-mC/EBPβ LAP were gifts from Jed Friedman (Addgene plasmids#66978 and #66979, respectively)^28^. pCF CREB was a gift from Marc Montiminy (Addgene plasmid #22968)^29^. For cloning of Pka-related constructs, cDNA synthesized from IEC-6 cells was used as the template for amplification of Prkaca, which was cloned into p3xFlag vectors. Its catalytic-site mutants (H87G, T196A, and H86G/T196A)^12^ were constructed using a site-directed mutagenesis kit (iNtRON Biotechnology, Korea) according to the manufacturer’s protocol. Cells were transiently transfected with 3 µg of plasmids using TurboFect (Fermentas Life Sciences, Ontario, Canada) in 60-mm dishes. After 24 h, the cells were harvested for analysis using quantitative RT-PCR or western blotting.

### Reverse transcription PCR and real-time PCR

mRNA expression was analyzed using RT-PCR and real-time PCR. Total RNA was extracted using the RNeasy Mini kit (GeneAll, Korea) and reverse-transcribed into cDNA using an iScript cDNA synthesis kit (Bio-Rad, Hercules, CA).The resulting cDNA was amplified using either a Maxime PCR PreMix kit (iNtRON Biotechnology) for conventional real-time PCR or the SYBR green method (iQ SYBR Green super mix, Bio-Rad) for real-time quantitative PCR. The primer sequences are listed in Table 1. For real-time PCR, triplicate reactions were performed for each analysis, and the cycle threshold (Ct) values of both test and reference genes (mouse sample, *Rpl13a*; human sample, *B2M*) were calculated using the ΔΔCt equation (2^−ΔΔCt^ method).

**Table 1:** Primers used for RT- and real-time PCR.

Gene	Primer Sequence (5′ to 3′, Forward, Reverse)
CLUH	AGGGGTTCGATTTCCTGAGT, CCAGGTATCGCATGTTGATG
PPARr	TTTTCAAGGGTGCCAGTTTC, AATCCTTGGCCCTCTGAGAT
C/EBPα	AGACATCAGCGCCTACATCG, GCTCCCGGGTAGTCAAAGTC
FABP4	AAACACCGAGATTTCCTTCAAA, CACGCCTTTCATAACACATTC
RPL13A	CCTGCTGCTCTCAAGGTTGTT, CGATAGTGCATCTTGGCCTTT
UCP1	GGCAAAAACAGAAGGATTGC, TAAGCCGGCTGAGATCTTGT
CIDEA	TGCTCTTCTGTATCGCCCAGT, GCCGTGTTAAGGAATCTGCTG
COX7A1	GTCTCCCAGGCTCTGGTCCG, CTGTACAGGACGTTGTCCATTC
GAPDH	AAGGGTCATCATCTCTGCCCC, GGGGGCCGAGTTGGGATAG
ACAT1	TGTAAAAGACGGGCTAACTGATG, TGTTCCTGCCGTGAGATATTCAT
ATP5A1	TGGGCCGTGTGGTTGAC, CGTCTGCGGGTCTTGGAA
HADHA	AAATCTGGGCCAACGACCAAA, CTTCCTGTGATATTCGTGTTGCT
PCCA	GGTTTTAGGGGATAAACATGGCA, CCATTGCTTGGCGAGTTTCA
β-ACTIN	GGACTTCGAGCAAGAGATGG, AGCACTGTGTTGGCGTACAG
PDHA1	GAAATGTGACCTTCATCGGCT, TGATCCGCCTTTAGCTCCATC
hCLUH	GGTTACACCATCTACAAGACGC, CTTGAGGTACTCGGAGCTTTC
hPPARr	CTCCGTGGATCTCTCCGTAA, CGACATTCAATTGCCATGAG
hALP	AAGCCGGTGCCTGGGTGGCCAT, ACAGGAGAGTCGCTTCAGAG
hB2M	AAGGACTGGTCTTTCTATCTCTTGTA, ACTATCTTGGGGTGTGACAAAGTC

### Western blotting

Western blotting was performed using the following primary antibodies: anti-CLUH (A301-764A; Bethyl Laboratories, Montgomery, TX), anti-PPAR-γ (SC-7273; Santa Cruz Biotechnology, Santa Cruz, CA), anti-FABP4 (#2120; Cell Signaling Technology, Beverly, MA), anti-Flag (F3165, Sigma-Aldrich) and anti-GAPDH (SC-25778, Santa Cruz Biotechnology). Immunoreactive proteins were detected using enhanced chemiluminescence (ECL) reagents (Santa Cruz Biotechnology).

### Promoter assay

A 1.4-kb genomic fragment encompassing bases −1414 to −1 from the translation start site (+1) of the mouse *Cluh* gene and a 1.4-kb genomic fragment encompassing bases −1391 to −36 from the translation start site (+1) of the human *CLUH* gene were cloned into the pGL3-Basic vector (Promega, WI). For the luciferase assay, NIH-3T3 cells on six-well plates were co-transfected with mock vector (pCDNA3 or p3XFlag) or plasmids encoding Creb or Prkaca (1 µg) plus the pRL Renilla luciferase control reporter vector (30 ng) and a promoter plasmid (1 µg). After 30 h, the cells were lysed with 0.2 ml passive lysis buffer (Promega). Luciferase activity was measured using a dual luciferase assay kit (Promega) according to the manufacturer’s protocol. Subsequently, the Renilla luciferase activity was measured. The activity of *Cluh* promoter-driven firefly luciferase gene in pGL3 cells was normalized to that of the corresponding Renilla luciferase to correct for between-sample differences in transfection efficiency. Each normalized promoter activity was plotted in terms of its relative fold-change compared to the normalized luciferase activity of cells transfected with the pGL3-basic vector and control vector (pCDNA3 or p3xFlag).

### Mitochondrial stress assay

The oxygen consumption rate (OCR) of siRNA-transfected differentiating cells was measured using a Seahorse XF24 analyzer and an XF assay kit (Seahorse Bioscience, North Billerica, MA). siRNA-transfected differentiating C3H10T1/2 cells (at day 3) were seeded at 3 × 10^4^ cells/well on an XF24 plate at 24 h before the assay. All samples were prepared in triplicate. On the day of the assay, the medium was changed to DMEM containing 584 mg/ml L-glutamine, without serum, glucose or bicarbonate, and the plates were incubated for 1 h in a non-CO_2_ incubator at 37 °C. The reagents for the assay were prepared using an XF Cell Mito Stress Test kit (Seahorse Bioscience, MA) according to the manufacturer’s protocol, and injection was performed according to a standard assay protocol (Port A: oligomycin; Port B: FCCP; and Port C: rotenone/antimycin A). Results were generated automatically using the Seahorse XF Mito Stress Test Reporter Generator.

### Reactive oxygen species measurement

Production of intracellular reactive oxygen species (ROS) was analyzed using flow cytometry. Cells were transfected with the appropriate siRNA, differentiated to adipocytes for 3 days, incubated with DCFDA (10 μM) for 30 min, and harvested using trypsin. Intracellular ROS levels were analyzed using the FL1 channel of a FACSCalibur flow cytometer (BD Biosciences).

### mRNA decay measurement

C3H10T1/2 cells were examined for adipocyte differentiation. The cells were transfected with siRNAs on day −1, differentiated into adipocytes or left undifferentiated on day 0, and treated with 2 μg/ml actinomycin D on day 6. The cells were harvested at 1, 2, 4 and 8 h after drug treatment.

### Statistical analyses

All data were analyzed as the mean ± SD. Statistical differences between two means were assessed using Student’s t test (unpaired, two-tailed). A p value of >0.05 was considered to be statistically non-significant (*n*.*s*.).

## Supplementary information

Supplementary
