# Supplementary material for: Cluh plays a pivotal role during adipogenesis by regulating the activity of mitochondria
Source: Sci Rep. 2019 May 2;9:6820. doi: 10.1038/s41598-019-43410-4 (PMC6497719; doi:10.1038/s41598-019-43410-4)
Supplement: Supplementary file 1 — Supplementary [file 41598_2019_43410_MOESM1_ESM.pdf]

Supplementary information:

Cluh plays a pivotal role during adipogenesis by regulating the activity of mitochondria

Eugene Cho [1], Wonhee Jung [1], Hyun-Yoo Joo[2], Eun-Ran Park[2], Mi-Yeon Kim[2], Su-Bin Kim[2], Kwang Seok Kim[2], Young Bin Lim[2], Kee Ho Lee[2], Hyun Jin Shin[1]

[1]Team of Radiation Convergence Research, Korea Institute of Radiological & Medical Sciences, Seoul, Korea. [2] Division of Radiation Biomedical Research, Korea Institute of Radiological & Medical Sciences, Seoul, Korea.

Supplementary Figure 1

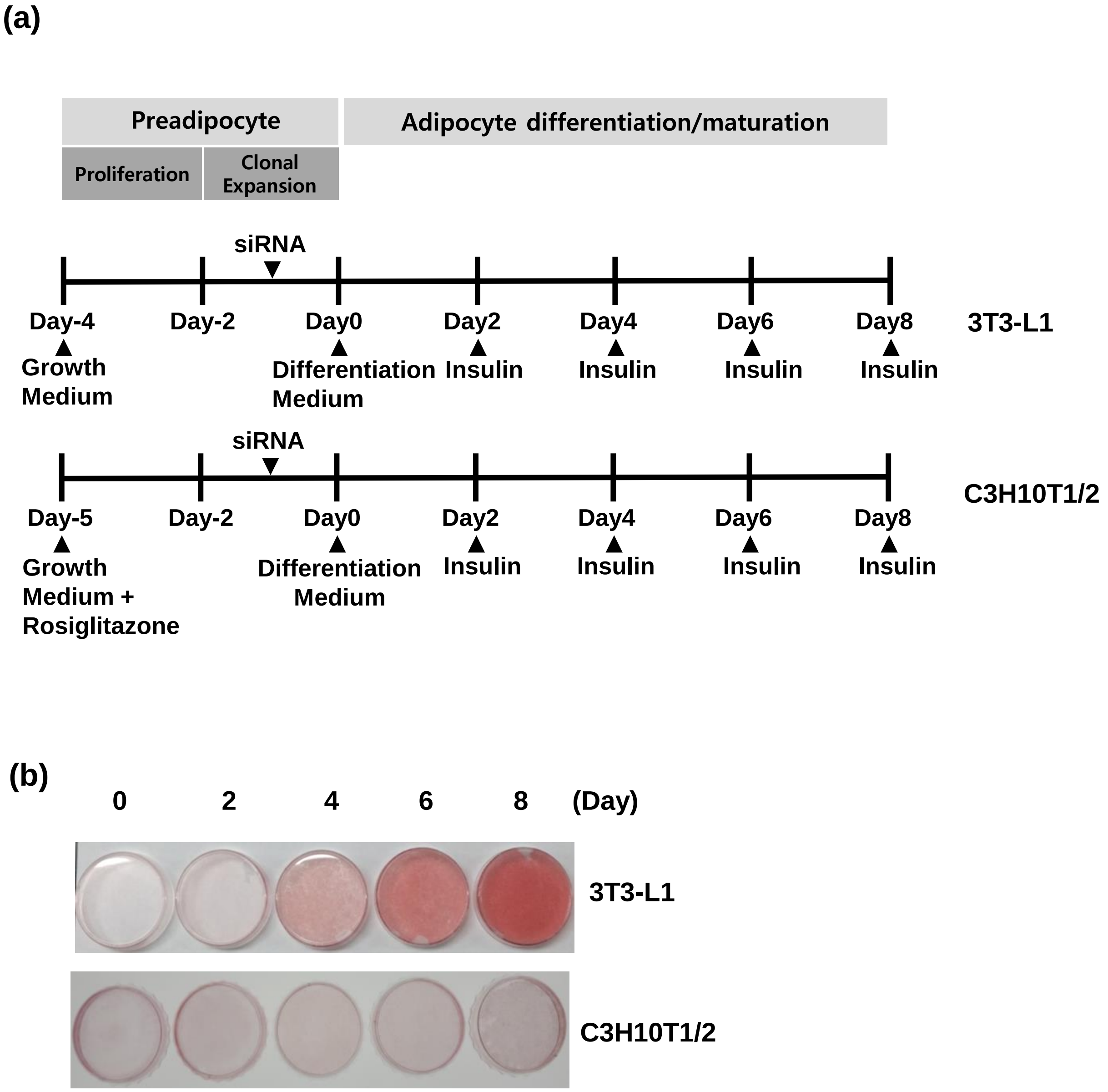

Supplementary Figure 1. (a) Schematic diagrams showing the differentiation protocols used for 3T3-L1 and C3H10T1/2 cells. **(b)** Differentiation was confirmed by staining with Oil Red O at 0, 2, 4, 6 and 8 days after adipocyte induction was initiated.

Supplementary Figure 2

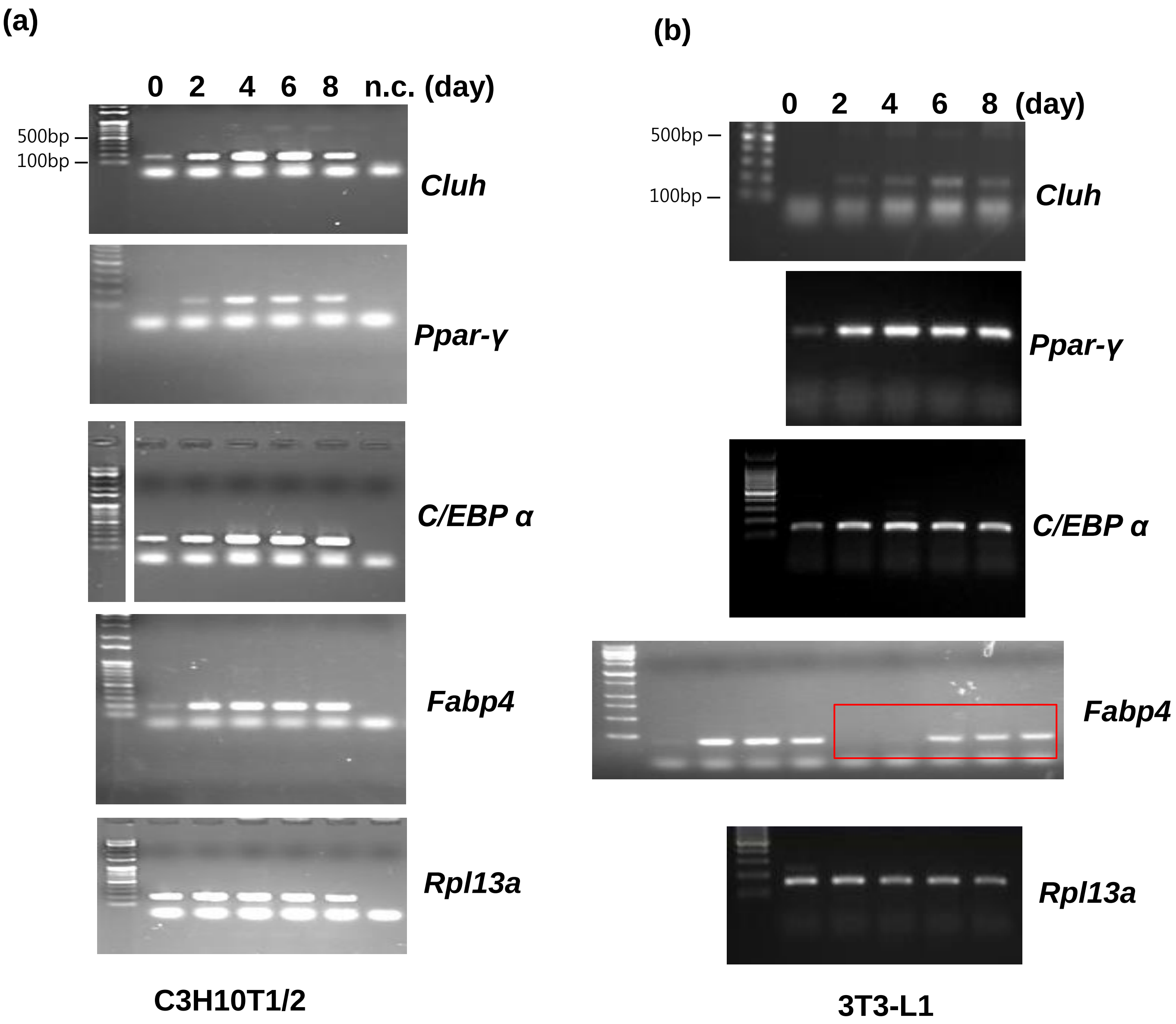

Supplementary Figure 2. Cluh mRNA is increased during adipogenesis. mRNA levels were assessed by RT-PCR analysis of differentiated C3H10T1/2 cells. **(a)** and 3T3-L1 cells **(b)**. n. c. ; no template control .

Supplementary Figure 3

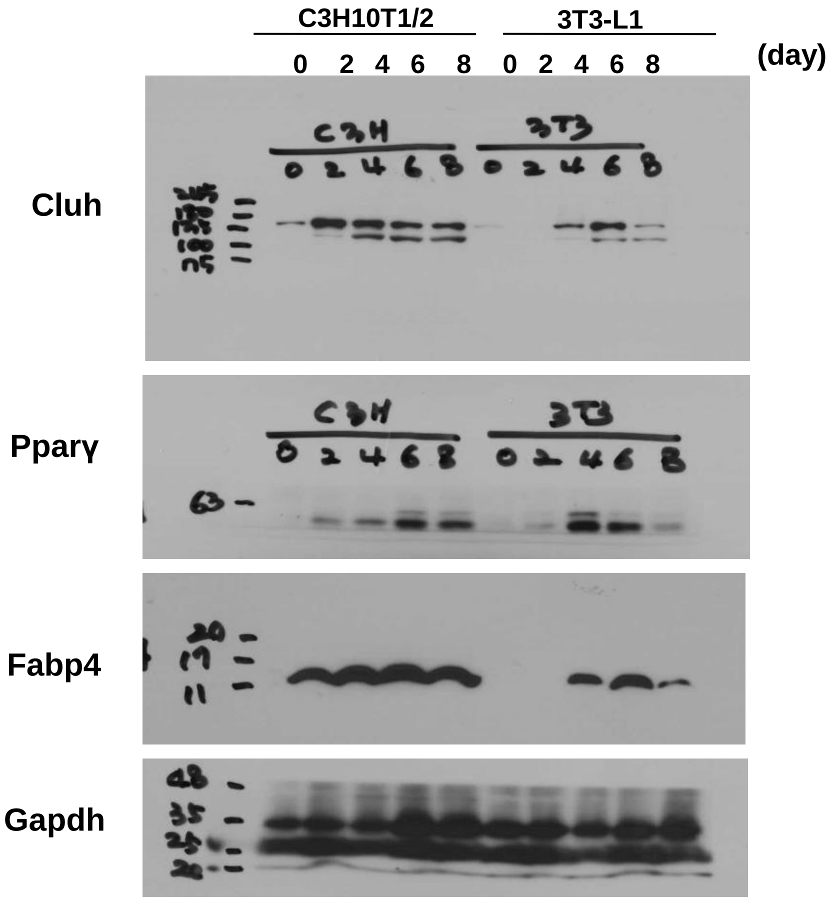

Supplementary Figure 3. Cluh is increased during adipogenesis. Protein levels were analyzed by Western blotting of lysates obtained from differentiated 3T3-L1 cells and C3H10T1/2 cells.

Supplementary Figure 4

(a)

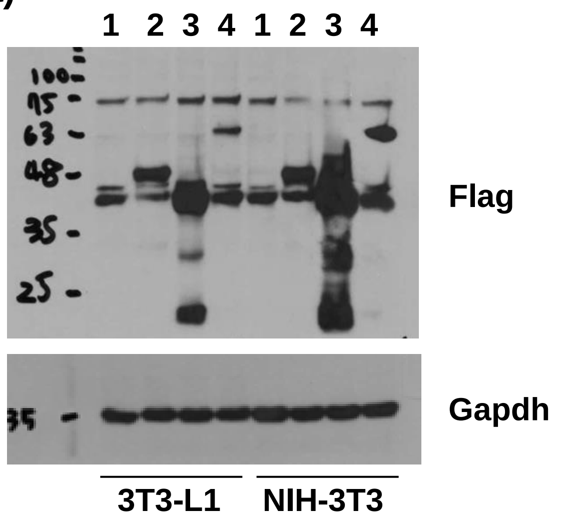

1. **pCDNA3**
2. **pCDNA3-Flag-Crebα (43Kda)**
3. **pCDNA3-Flag-Crebβ (31Kda)**
4. **pCDNA3-Flag-Ppar-γ (65Kda)**

(b)

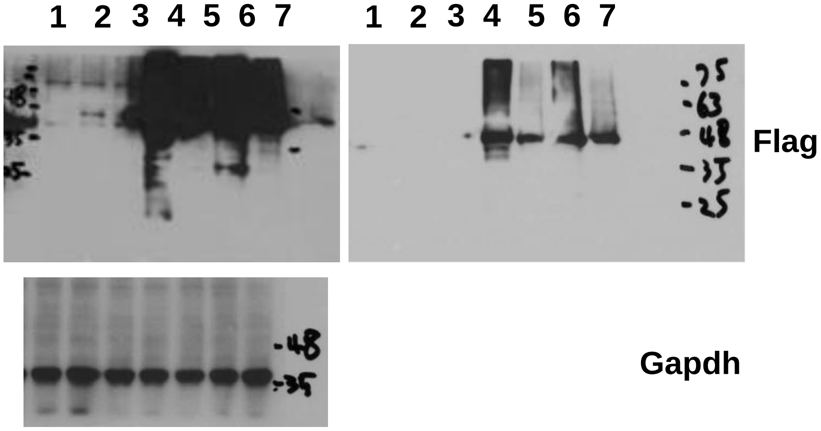

1. **pCDNA3**
2. **pCF-Creb (45 Kda)**
3. **p3xFlag**
4. **p3xFlag-Prkaca (40 Kda)**
5. **p3xFlag-Prkaca H87G**
6. **p3xFlag-Prkaca T196A**
7. **p3xFlag-Prkaca H87G/T196A**

(c)

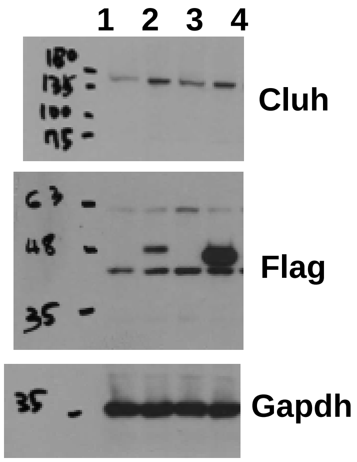

1. **pCDNA3**
2. **pCF-Creb (45 Kda)**
3. **p3xFlag**
4. **p3xFlag-Prkaca (40 Kda)**

Supplementary Figure 4. The expression of Flag-tagged proteins was confirmed in plasmid-transfected NIH-3T3 cells **(a, b)** and 3T3-L1 cells **(a, c**) by Western blot analysis using a Flag antibody. The expected size and transfected plasmids are listed for each sample. The increase of Cluh protein by CREB and PKA were confirmed by western blot analysis at 3T3-L1 cells **(c)**.

Supplementary Figure 5

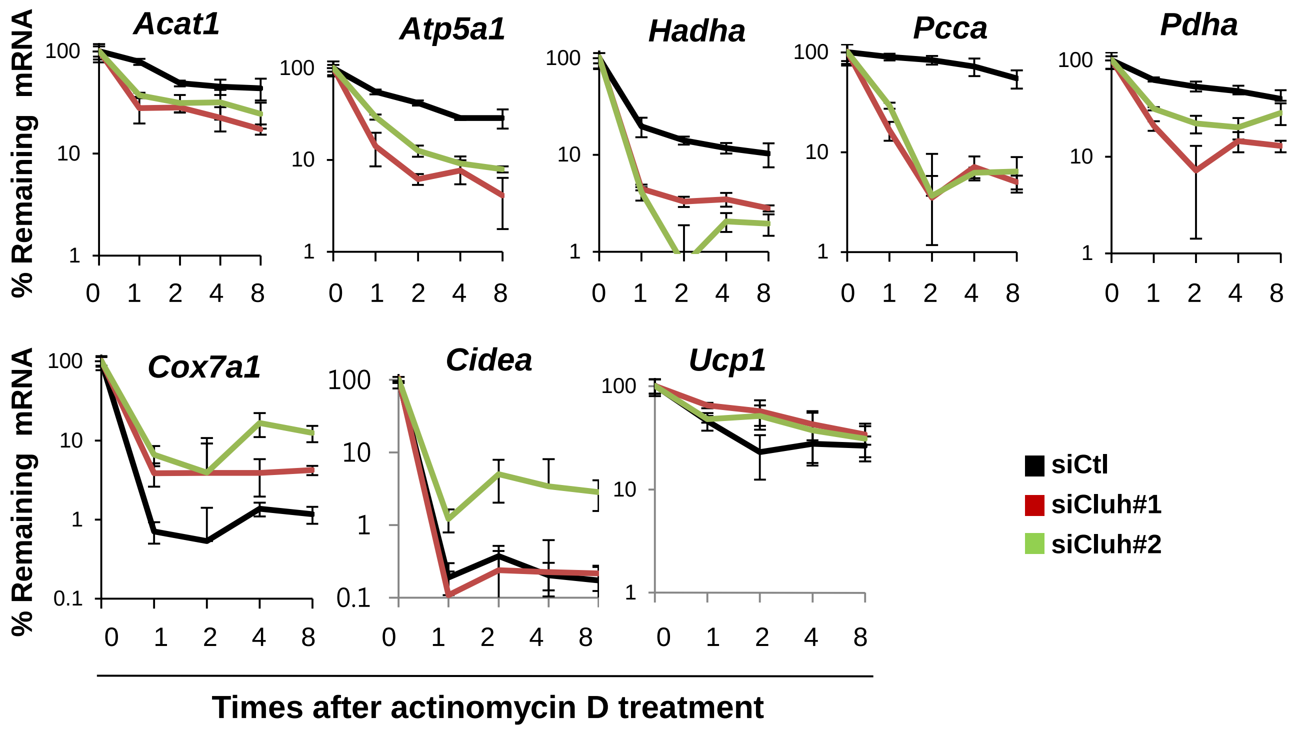

Supplementary Figure 5. The effect of Cluh depletion on RNA stability. siRNA-transfected and differentiated C3H10T1/2 cells were treated with actinomycin D (2 μg/ml) and harvested at the indicated times. The data represent those presented in Figures 5b and d, transformed with log scale.
